# A bioinformatic pipeline for simulating viral integration data

**DOI:** 10.1016/j.dib.2022.108161

**Published:** 2022-04-10

**Authors:** Suzanne Scott, Susanna Grigson, Felix Hartkopf, Claus V. Hallwirth, Ian E. Alexander, Denis C. Bauer, Laurence O.W. Wilson

**Affiliations:** a Australian e-Health Research Centre, Commonwealth Scientific and Industrial Research Organisation, North Ryde, Australia; b Gene Therapy Research Unit, Children's Medical Research Institute, Westmead, Australia; c The Sydney Children's Hospitals Network, Faculty of Medicine and Health, The University of Sydney, Westmead, Australia; d College of Science and Engineering, Flinders University, Adelaide, Australia; e Department of Mathematics and Computer Science, Freie Universität Berlin, Berlin, Germany; f Discipline of Child and Adolescent Health, Faculty of Medicine and Health, The University of Sydney, Sydney, NSW, SA; g Macquarie University, Department of Biomedical Sciences, Faculty of Medicine and Health Science, Macquarie Park, SA; h Macquarie University, Applied BioSciences, Faculty of Science and Engineering, Macquarie Park, SA

**Keywords:** *In silico*, Virus, Gene therapy, Vector, Integration

## Abstract

Viral integration is a complex biological process, and it is useful to have a reference integration dataset with known properties to compare experimental data against, or for comparing with the results from computational tools that detect integration. To generate these data, we developed a pipeline for simulating integrations of a viral or vector genome into a host genome. Our method reproduces more complex characteristics of vector and viral integration, including integration of sub-genomic fragments, structural variation of the integrated genomes, and deletions from the host genome at the integration site. Our method [1] takes the form of a snakemake [2] pipeline, consisting of a Python [3] script using the Biopython [4] module that simulates integrations of a viral reference into a host reference. This produces a reference containing integrations, from which sequencing reads are simulated using ART [5]. The IDs of the reads crossing integration junctions are then annotated using another python script to produce the final output, consisting of the simulated reads and a table of the locations of those integrations and the reads crossing each integration junction. To illustrate our method, we provide simulated reads, integration locations, as well as the code required to simulate integrations using any virus and host reference. This simulation method was used to investigate the performance of viral integration tools in our research [6].

## Specifications Table

**Table:** 

Subject	Computational Biology
Specific subject area	Bioinformatics; Simulation
Type of data	Code DNA sequence (fasta) Next-generation sequencing reads (fastq) Table
How the data were acquired	Our simulation pipeline was developed using snakemake 5.27 [2], Python 3.7 [3], biopython 1.76 [4], Pysam 0.16 [7,8], NumPy [9], Pandas 1.0 [10], SciPy 1.5 [11] and ART 2016.06.05 [5]. The user may make use of either Conda [12] or Singularity [13] to supply these dependencies automatically (via snakemake). Our pipeline works on Linux and MacOS, but has not been tested on Windows. In our example data, we simulated integrations of AAV2 (acquired from GenBank, accession NC_001401.2) into human chromosome 1 (GenBank, accession NC_000001.11). The example data were generated on a Dell PowerEdge C6525 server with 512 GB of RAM and dual AMD EPYC 7543 32-Core Processors running at 2.8 GHz with 256 MB cache.
Data format	Raw, Simulated
Description of data collection	The simulation pipeline begins by simulating integration by taking pieces of the viral reference and inserting them into the host reference, keeping track of which parts of the viral reference were integrated and where in the host genome the integrations occurred. This step is carried out by a Python script that outputs a file in fasta format containing the host reference with integrated viral sequences and a table containing the location of the integrations. The properties of these integrations can be adjusted by setting the number of integrations, the minimum distance between adjacent integrations, the probability that the whole viral genome will be integrated (or a sub-genomic fragment), the minimum and maximum length of the sub-genomic fragments (if appropriate), the probability that the integrated genome will contain a rearrangement or deletion, the probability of a gap or overlap at the host/virus junctions, and the probability of a deletion from the host at each integration site. After integration simulation, reads are generated using ART [5]. At this step, the user can specify a read length (we simulate paired-end reads), fold-coverage, mean fragment length and standard deviation, and a sequencing system from which an error profile is derived.
	Next, the reads that cross each integration junction are identified by a Python script, and the table of integration locations is updated with this information. Finally, a file containing the locations in the host of the integration junctions which are crossed by at least one read is output in BED format. The primary outputs of the pipeline are the simulated reads (fastq format), the table containing information about each integration, and the table containing the locations of each integration with at least one supporting read in BED format.
Data source location	AAV2 and human chr1 references (GenBank) • *Institution:* National Centre for Biotechnology Information • *City/Town/Region: Bethesda MD* • *Country: USA*
Data accessibility	Repository name: GitHub (code only) Data identification number: https://doi.org/10.5281/zenodo.6403449 Direct URL to data: https://github.com/aehrc/vector-integration-simulation-pipeline Repository name: CSIRO data access portal (code and example dataset) Data identification number: https://doi.org/10.25919/m529-q062 Direct URL to data: https://data.csiro.au/collection/csiro:53212
Related research article	S. Scott, C.V. Hallwirth, F. Hartkopf, S. Grigson, Y. Jain, I.E. Alexander, D.C. Bauer, L.O.W. Wilson, Isling: A Tool for Detecting Integration of Wild-Type Viruses and Clinical Vectors, Journal of Molecular Biology. (2021) 167,408. https://doi.org/10.1016/j.jmb.2021.167408.

## Value of the Data

• Having a way to simulate integrations is useful when comparing software that detect integrations, by creating a ‘ground truth’ against which outputs can be compared
• This pipeline has been used to validate software for the detection of viral integrations [6]
• It may also be useful for researches investigating virus or vector integrations, to compare their results against simulated integrations

## 1 Data Description

**Fig. 1:**
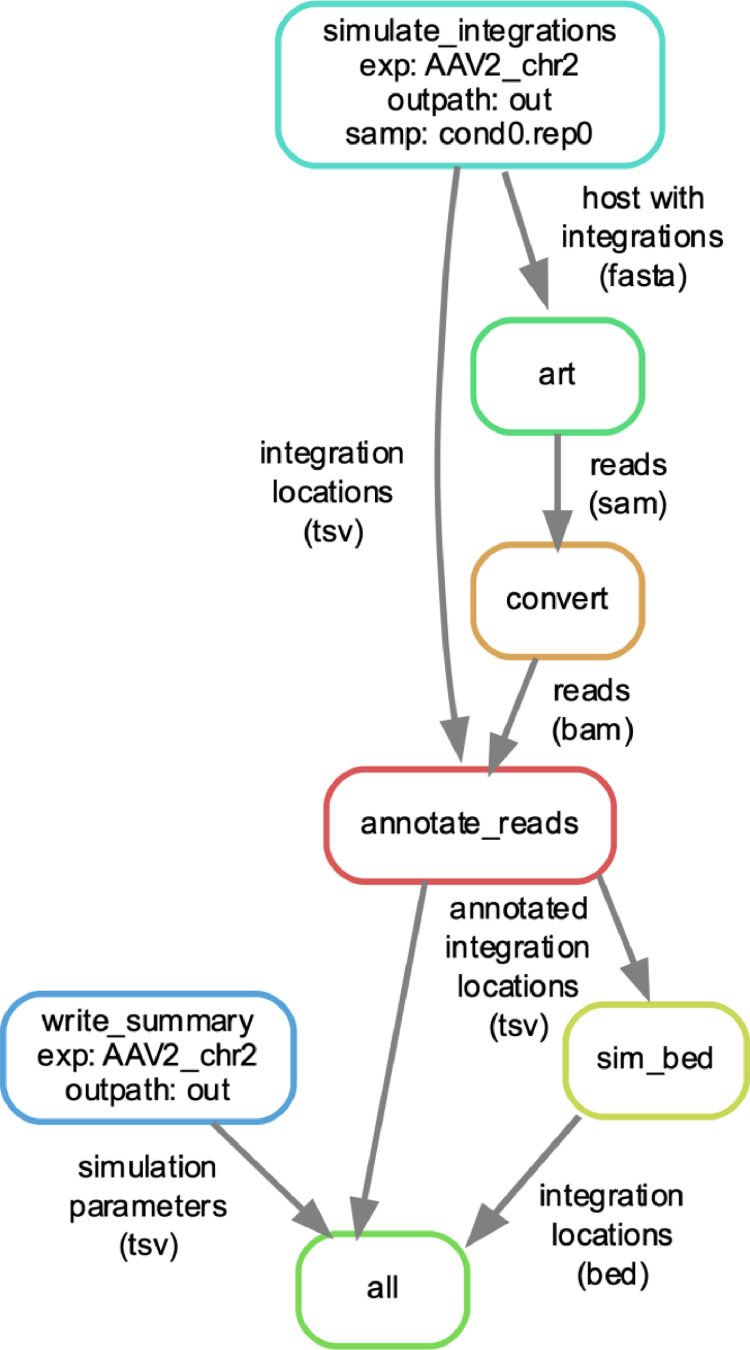
The workflow for simulating the example data. Boxes represent steps in simulating the data, and arrows represent files which are output by one step and input into the next. First, integrations are simulated (simulate_integrations, aqua), and then reads are generated using ART (green). The resulting SAM file is sorted and coverted to BAM format (orange) and then the reads crossing each integration are annotated (red). The locations of the integrations across which reads cross are then output in BED3 format (yellow). A summary table of the integration simulation parameters is also written to a file (blue). The resulting summary, fastq files, and tables containing the locations of the integrations are the primary output of the pipeline (all, light green. This figure was generated using snakemake [2] and dot [14].

Our data consist code to simulate integrations and create tables of their properties, consisting of a snakemake workflow and several Python scripts, as well as an example dataset to illustrate the method [1]. The steps in the simulation pipeline are illustrated in Fig. 1.

The simulated integrations can be tailored to the integration behaviour of a particular virus or vector. Here, we simulate the integration of wild-type AAV2 (GenBank NC_001401.2) into human chromosome 1 (GRCh38, GenBank NC_000001.11), with 100 integrations per replicate. Integrations of AAV often involve sub-genomic fragments (rather than the whole virus) [15], so the probability of a sub-genomic fragment being integrated was 0.5, with a minimum length of 50 bp. Structural variation has also been observed in integrated AAV genomes [15,16], so the probabilities of rearrangement and deletion were both 0.1, and the mean of the Poisson distribution from which the number of pieces into which the viral fragment was split during rearrangement or deletion was 1. There is also frequently a gap (of bases that appear to come from neither host nor virus) or overlap (microhomology between host and vector) at the host/virus junction [16], so the probabilities of an overlap or gap each junction were both 0.2, with a mean length of 1 bases involved in each junction. Finally, deletions from the host genome can occur at integration sites [16], so the probability of a deletion occurring at each integration site was 0.2. If a deletion occurred, its length was drawn from a Poisson distribution with a mean of 20 bp.

The example results obtained are hosted on the CSIRO data access portal (https://doi.org/10.25919/m529-q062). The output files are in the out/AAV2_chr1 directory:

• simulation_summary.tsv: Each of the output files are have the prefix ‘condX.repY’ for condition number X and replicate number Y of that condition. This file contains a table of the parameters used for each condition and replicate
• sim_ints: this folder contains information about the simulated integrations:
  ○ Files ending in ‘.int-info.tsv’ contain a table with the location and properties of each simulated integration in the original host reference, and the newly constructed reference with integrations. Generally, the column ‘hPos’ is of interest – this is the location of the integrations in the original host reference.
  ○ Files ending in ‘.int-info.annotated.tsv’ contain the same information as the previous file, but additionally has the reads that cross each integration junction annotated.
  ○ Files ending in ‘int-info.bed’ contain the location of the integration junctions with supporting reads in the original host genome, in BED3 format.
  ○ Files ending in ‘epi‑info.tsv’ contain a table with information about the episomes that were included in the simulation.
• sim_reads: this folder contains the simulated reads
  ○ The files ending in ‘1.fq’ and ‘2.fq’ contain the simulated read 1 and read 2 sequences and qualities, respectively.
  ○ Files ending in ‘sorted.bam’ contain these reads aligned to the reference containing integrations (which is different to the original host reference), and the index of these files end in ‘sorted.bam.bai’. The alignment files are coordinate-sorted.

The rest of the files are part of the pipeline for simulation:

• ‘README.md’: A readme containing information about the pipeline
• ‘Snakefile’: The file specifying the snakemake workflow for simulating integrations and reads
• ‘Dockerfile’: A dockerfile for creating a docker container for the workflow.
• ‘config/simulation.yml’: The config file used for creating the example data
• ‘references/AAV2.fa’, ‘reference/chr1/fa’: The references used for creating the example data
• ‘scripts/’: The scripts used for simulating integrations
• ‘snakemake_rules’: A directory containing the rules for running the snakemake workflow

## 2 Experimental Design, Materials and Methods

The first step in simulating integrations is reading the config file and creating the conditions. Each combination of the parameters specified in the config file is one condition, and there is one or more replicates of each condition (with a different random seed). The conditions used for simulation are written to a tab-separated table in a file called ‘simulation_summary.tsv’ (see above).

Next, a reference containing integrations is created. This is achieved using the Python3 script ‘scripts/insert_virus.py’, which randomly selects a host chromosome and viral reference, and then adds the viral sequence to the host chromosome at a random position. This process is repeated to produce the number of integrations set by the *n_ints* parameter.

Depending on the parameters used, this might always be the whole virus (if *p_whole* is 1), or a randomly-selected sub-genomic fragment. The minimum and maximum length of the integrations can be controlled by setting the max*_len* and min*_len* parameters in the config file. Structural variation can be simulated by setting the *p_rearrange* and *p_delete* paramters to a number greater than 0 (and less than or equal to 1).

If the integrated viral fragment is to be rearranged or deleted, it is first split into a number of smaller pieces. The number of pieces is an integer drawn from a Poisson distribution, with a mean set by the parameter *lambda_split*. If the viral fragment is to be rearranged, two of the pieces are swapped before integration, and if the viral fragment is to contain a deletion, one of the pieces is removed.

Then, the junctions between each end of the viral fragment and the host chromosome are created – these are either a gap (containing randomly selected bases), an ‘overlap’ where the junction contains homology between the host and vector, or a ‘clean’ junction where the host chromosome runs straight into the viral sequence. The probability of obtaining each kind of junction is determined by the parameters *p_gap* and *p_overlap*, and the length of the junction is an integer drawn from a Poisson distribution, with a mean set by the parameter *lambda_junction*.

A deletion from the host at the integration site can also be simulated, and the probability of this event is set by the parameter *p_host_deletion*. If a deletion occurs, it's length draws from a Poisson distribution with a mean set by the parameter *lambda_host_deletion*.

Episomal (non-integrated) viral sequences can also be included in the reference by specifying a number of episomes to include (*epi_num*). If these are included, these may be subject to rearrangement or deletion, depending on the values of *p_rearrange* and *p_delete*.

After this step, a new reference containing integrations and episomal sequences is created, as well as a table of information about each integration.

Next, the reference containing integrations is use to simulate paired-end short sequencing reads. This step is performed by *art_illumina* [5], and the user can specify the read length, fold coverage, mean and standard deviation of the fragment length and sequencing system.

After reads are simulated, the reads crossing each integration junction are identified, and a file containing the location of integrations in the original host genome is generated.

## Ethics Statements

The use of human data was approved by the CSIRO health and medical research ethics committee (**2019_032_LR**).

## CRediT Author Statement

**Suzanne Scott:** Conceptualization, Methodology, Software, Investigation, Writing – Original draft**.  Susanna Grigson:** Software**. Felix Hartkopf:** Software. **Claus Hallwirth:** Conceptualization. **Ian Alexander:** Conceptualization, supervision. **Denis Bauer:** Writing – Review and Editing, Supervision. **Laurence Wilson:** Conceptualization, Supervision, Writing – Review and Editing

## Declaration of Competing Interest

The authors declare that they have no known competing financial interests or personal relationships that could have appeared to influence the work reported in this paper.

## Data Availability

Pipeline for simulating integrations (Original data) (CSIRO Data Access Porta).
